# Supplementary material for: Identifying essential genes in genome-scale metabolic models of consensus molecular subtypes of colorectal cancer
Source: PLoS One. 2023 May 19;18(5):e0286032. doi: 10.1371/journal.pone.0286032 (PMC10198572; doi:10.1371/journal.pone.0286032)
Supplement: S1 Text — The source programs for identifying essential targets are coded using the General Algebraic Modeling System. (PDF) [file pone.0286032.s007.pdf]

Computational Procedures for Solving ACTD Problem

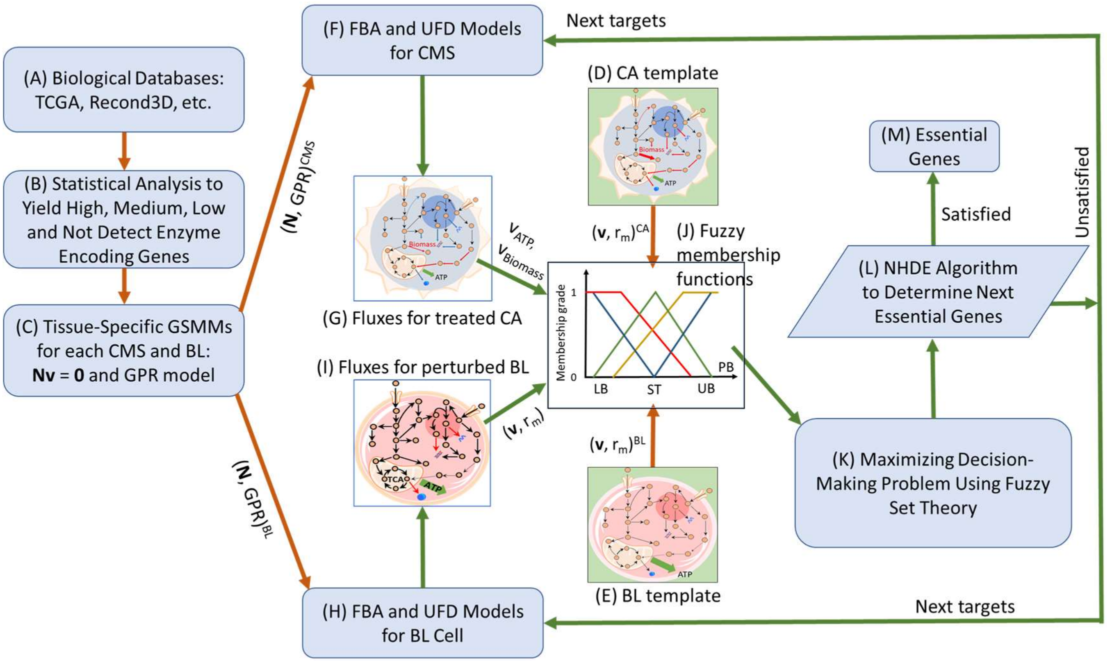

Figure S1. ACTD Framework for identifying essential anticancer genes.

The preliminary steps for solving ACTD problem are to reconstruct each tissue-specific genome-scale metabolic models for each CMS and healthy (HT) cells, and then to provide the templates for each CMS and HT models. Here, we denote the CMS as CA model. The preliminary steps are explained as the following three procedures.

1. We download RNA-seq expression for cancerous (CA) and healthy cells (HT to reconstruct tissue-specific GSMM and the corresponding GPR association for each CMS and HT cells, respectively (Step (A)-(C) in Figure 1).

2. We build the constraint-based (CMB) model for CA and HT models as follows.

$$
\begin{cases}
\text{CA model:} \\
\text{FBA problem:} \qquad\qquad\qquad\qquad \text{UFD problem:} \\
\begin{cases}
\max\limits_{\mathbf{v}_{f/b}} obj = v_{biomass} \\
\text{subject to} \\
\mathbf{N}^{CA}\left(\mathbf{v}_f - \mathbf{v}_b\right) = \mathbf{0} \\
v_{f/b,j}^{LB} \le v_{f/b,j} \le v_{f/b,j}^{UB},\ j \in \Omega^{CA}
\end{cases}
\begin{cases}
\min\limits_{\mathbf{v}_{f/b}} \sum\limits_{k\in\Omega^{Int}} c_k^{CA}\left(\left(v_{f,k}\right)^2 + \left(v_{b,k}\right)^2\right) \\
\text{subject to} \\
\mathbf{N}^{CA}\left(\mathbf{v}_f - \mathbf{v}_b\right) = \mathbf{0} \\
v_{f/b,j}^{LB} \le v_{f/b,j} \le v_{f/b,j}^{UB},\ j \in \Omega^{CA} \\
v_{biomass} \ge v_{biomass}^{*}
\end{cases} \\
\text{HT model:} \\
\text{FBA problem:} \qquad\qquad\qquad\qquad \text{UFD problem:} \\
\begin{cases}
\max\limits_{\mathbf{v}_{f/b}} obj = v_{ATP} \\
\text{subject to} \\
\mathbf{N}^{HT}\left(\mathbf{v}_f - \mathbf{v}_b\right) = \mathbf{0} \\
v_{f/b,j}^{LB} \le v_{f/b,j} \le v_{f/b,j}^{UB},\ j \in \Omega^{HT}
\end{cases}
\begin{cases}
\min\limits_{\mathbf{v}_{f/b}} \sum\limits_{k\in\Omega^{Int}} c_k^{HT}\left(\left(v_{f,k}\right)^2 + \left(v_{b,k}\right)^2\right) \\
\text{subject to} \\
\mathbf{N}^{HT}\left(\mathbf{v}_f - \mathbf{v}_b\right) = \mathbf{0} \\
v_{f/b,j}^{LB} \le v_{f/b,j} \le v_{f/b,j}^{UB},\ j \in \Omega^{HT} \\
v_{ATP} \ge v_{ATP}^{*}
\end{cases}
\end{cases}
\tag{S1}
$$

where the stochiometric matrices, $\mathbf{N}^{CA}$ and $\mathbf{N}^{HT}$, for CA and HT models are reconstructed using Step (A)-(C) in Figure 1. The RNA-seq expressions for CA and HT cells and GPR associations in Recon3D are used to set the weighting factors, c_k^{CA} and c_k^{HT}, for UFD problems; the four groups of confidence reactions are assigned as follows:

$$
c_k^{CA/HT} = \begin{cases}
\frac{1}{4},\ k \in \text{high confidence} \\
\frac{1}{2},\ k \in \text{medium confidence} \\
\frac{3}{4},\ k \in \text{negativec confidence} \\
1,\ k \in \text{other confidence or non-gene-expression}
\end{cases}
\tag{S2}
$$

3. We have to provide CA and HT templates (Figure 1D and 1E) for the ACTD framework for identifying anticancer targets. Clinical data of the fluxes and metabolite flow can be used as the CA and HT templates. However, genome-scale clinical data are currently not available. We use Eq.(S1) to compute optimal fluxes and metabolite flow rates for CA and HT cells to provide as the templates. The computational procedures are expressed in Fig. S2

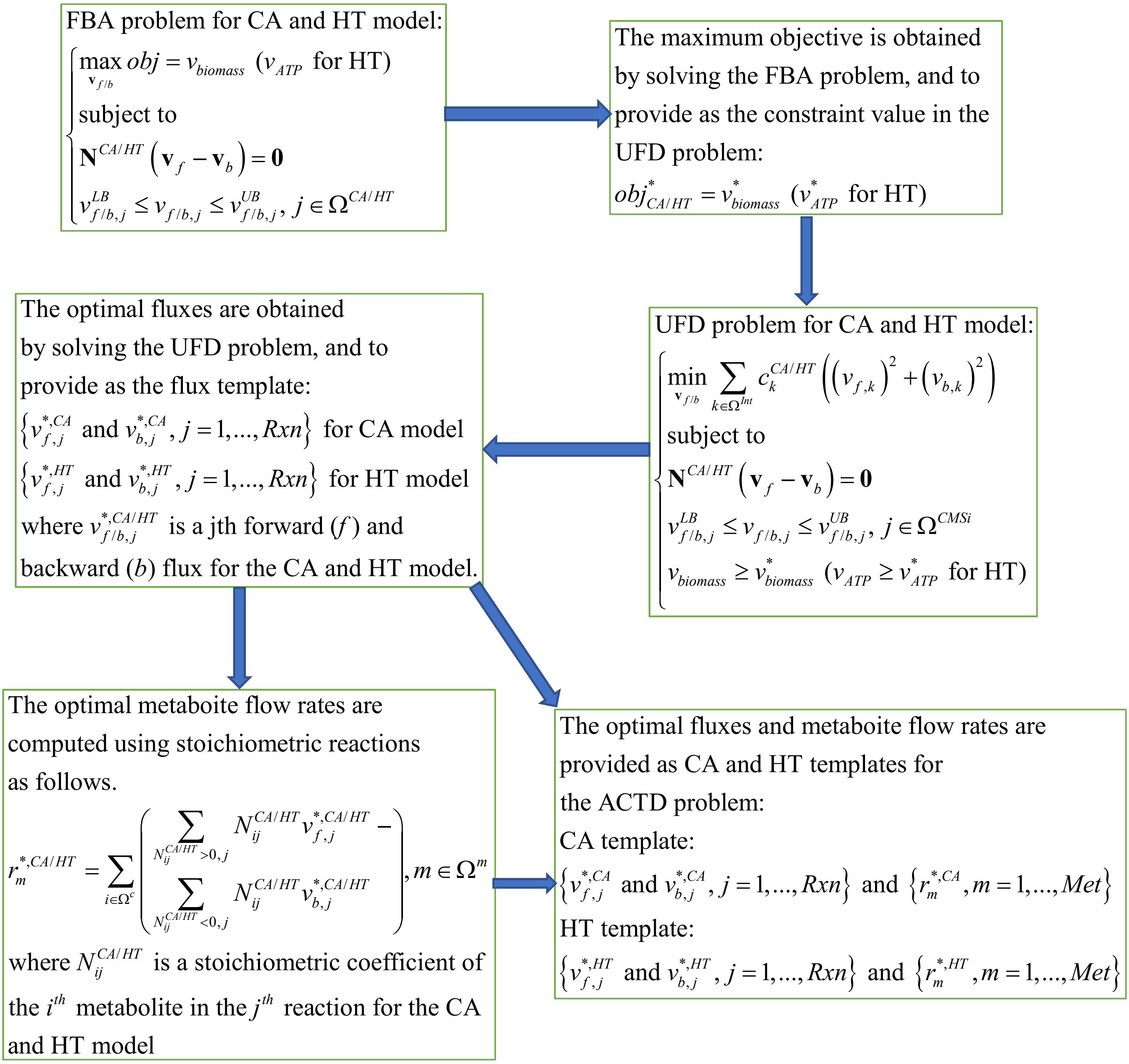

Figure S2. Computational procedures to obtain the optimal fluxes and metabolite flow rates provided as CA and HT templates.

4. The ACTD problem is transformed into a maximizing decision-making (MDM) problem through fuzzy set theory as follows.

$$\begin{cases} \max\limits_{\mathbf{z}} \eta_D = \max\limits_{\mathbf{z}}\left(\eta_{TR} + \min\{\eta_{TR}, \eta_{CV}, \eta_{MD}\}\right)/2 \\ \text{subject to inner optimization problems} \\ \text{1. FBA and UFD problems for treated CA model} \\ \text{2. FBA and UFD problems for perturbed HT model} \end{cases} \quad \text{(S3)}$$

5. We apply the nested hybrid differential evolution (NHDE) algorithm to solve the MDM problem that mimics a wet-lab to identify anticancer targets as shown in Figure 1D to 1 M. The key iterative procedures of the NHDE algorithm are mutation, crossover and fitness evaluation (Table S1), that are introduced in the below section. Here, we explain how to compute a decision-making criterion (referred to as fitness in the NHDE algorithm) for each generated gene.

6. The NHDE algorithm is a parallel direct search algorithm that generates a population of target genes, and then each target is applied to find its corresponding optimal solution. A gene can regulate one or several reactions to restrict the lower and upper bounds of fluxes as in Eq.(S1) to express as follows.

$$\begin{cases} \text{Treated CA (TR) model:} \\ \text{FBA problem:} \qquad\qquad \text{UFD problem:} \\ \begin{cases} \max\limits_{\mathbf{v}_{f/b}} obj = v_{biomass} \\ \text{subject to} \\ \mathbf{N}^{CA}\left(\mathbf{v}_f - \mathbf{v}_b\right) = \mathbf{0} \\ v_{f/b,i} = 0,\ z_i \in \Omega^z \\ v_{f/b,j}^{LB} \le v_{f/b,j} \le v_{f/b,j}^{UB},\ j \notin \Omega^z \end{cases} \quad \begin{cases} \min\limits_{\mathbf{v}_{f/b}} \sum\limits_{k\in\Omega^{Int}} c_k^{CA}\left(\left(v_{f,k}\right)^2 + \left(v_{b,k}\right)^2\right) \\ \text{subject to} \\ \mathbf{N}^{CA}\left(\mathbf{v}_f - \mathbf{v}_b\right) = \mathbf{0} \\ v_{f/b,i} = 0,\ z_i \in \Omega^z \\ v_{f/b,j}^{LB} \le v_{f/b,j} \le v_{f/b,j}^{UB},\ j \notin \Omega^z \\ v_{biomass} \ge v_{biomass}^{*} \end{cases} \\ \text{Perturbed HT (PH) model:} \\ \text{FBA problem:} \qquad\qquad \text{UFD problem:} \\ \begin{cases} \max\limits_{\mathbf{v}_{f/b}} obj = v_{ATP} \\ \text{subject to} \\ \mathbf{N}^{HT}\left(\mathbf{v}_f - \mathbf{v}_b\right) = \mathbf{0} \\ v_{f/b,i} = 0,\ z_i \in \Omega^z \\ v_{f/b,j}^{LB} \le v_{f/b,j} \le v_{f/b,j}^{UB},\ j \notin \Omega^z \end{cases} \quad \begin{cases} \min\limits_{\mathbf{v}_{f/b}} \sum\limits_{k\in\Omega^{Int}} c_k^{HT}\left(\left(v_{f,k}\right)^2 + \left(v_{b,k}\right)^2\right) \\ \text{subject to} \\ \mathbf{N}^{HT}\left(\mathbf{v}_f - \mathbf{v}_b\right) = \mathbf{0} \\ v_{f/b,i} = 0,\ z_i \in \Omega^z \\ v_{f/b,j}^{LB} \le v_{f/b,j} \le v_{f/b,j}^{UB},\ j \notin \Omega^z \\ v_{ATP} \ge v_{ATP}^{*} \end{cases} \end{cases}$$

where z_i is the ith gene to be deleted (S4)

7. Using the similar procedures in Fig. S2, the optimal fluxes and metabolite flow rates for TR and PH model of the ith gene are obtained as follows.

The optimal fluxes and metaboite flow rates for TR and PH model of the ith gene are applied to evaluate membership functions in the MDM problem:

TR model:

$$\left\{v_{f,j}^{i,TR} \text{ and } v_{b,j}^{i,TR}, j=1,\ldots,Rxn\right\} \text{ and } \left\{r_m^{i,TR}, m=1,\ldots,Met\right\}$$

PH model:

$$\left\{v_{f,j}^{i,PH} \text{ and } v_{b,j}^{i,PH}, j=1,\ldots,Rxn\right\} \text{ and } \left\{r_m^{i,PH}, m=1,\ldots,Met\right\} \qquad \text{(S5)}$$

8. The optimal solutions for all target genes obtained from Eq.(S5) are applied to compute membership function grades for fuzzy minimization, maximization, dissimilarity and similarity to yield the cell mortality grade η_{TR}, cell viability grade η_{CV}, and metabolic deviation grade η_{MD} as follows.

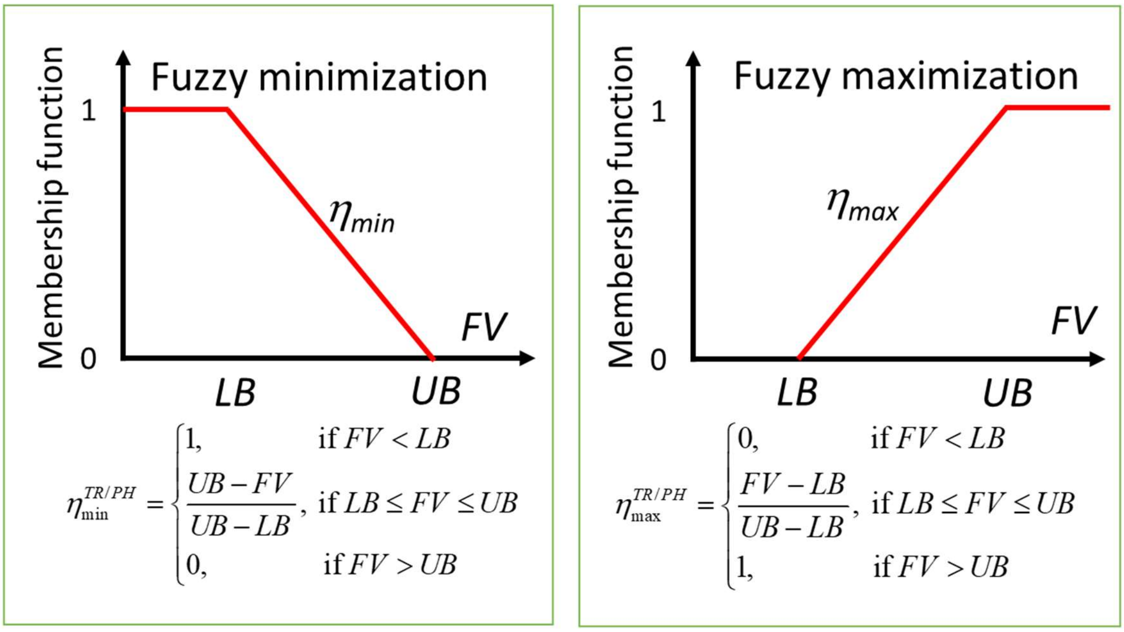

The flux *FV* is obtained by solving Eq.(S5), and the lower bound *LB* and the upper bound *UB* are provided from *CA* and *HT* templates from Eq.(S1).

Figure S3. One-side member function to attribute fuzzy minimization and maximization

The cell mortality grade η_{TR} for treated CA model and cell viability grade η_{CV} for perturbed HT model are evaluated by the mean-min operation, and expressed as follows.

Cell mortality grade:

$$\eta_{TR} = \left(\left(\eta^{TR}_{\min,biomass} + \eta^{TR}_{\min,ATP}\right)/2 + \min\left\{\eta^{TR}_{\min,biomass}, \eta^{TR}_{\min,ATP}\right\}\right)/2$$

Cell viability grade:

$$\eta_{CV} = \left(\left(\eta^{PH}_{\min,biomass} + \eta^{PH}_{\max,ATP}\right)/2 + \min\left\{\eta^{PH}_{\min,biomass}, \eta^{PH}_{\max,ATP}\right\}\right)/2$$

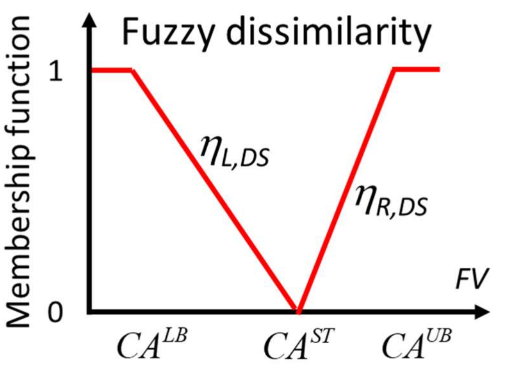

$$\eta_{L,DS}(FV) = \begin{cases} 1, & \text{if } FV < CA^{LB} \\ \dfrac{CA^{ST} - FV}{CA^{ST} - CA^{LB}}, & \text{if } CA^{LB} \le FV \le CA^{ST} \\ 0, & \text{if } FV > CA^{ST} \end{cases}$$

$$\eta_{R,DS}(FV) = \begin{cases} 0, & \text{if } FV < CA^{ST} \\ \dfrac{FV - CA^{ST}}{CA^{UB} - CA^{ST}}, & \text{if } CA^{ST} \le FV \le CA^{UB} \\ 1, & \text{if } FV > CA^{UB} \end{cases}$$

$$\eta_{DS} = \min\left\{\max\left\{\eta_{L,DS}, \eta_{R,DS}, 0\right\}, 1\right\}$$

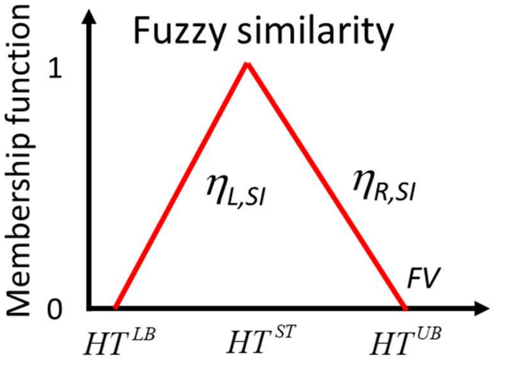

$$\eta_{L,SI} = \begin{cases} 0, & \text{if } FV < HT^{ST} \\ \dfrac{FV - HT^{LB}}{HT^{ST} - HT^{LB}}, & \text{if } HT^{LB} \le FV \le HT^{ST} \\ 1, & \text{if } FV > HT^{ST} \end{cases}$$

$$\eta_{R,SI} = \begin{cases} 1, & \text{if } FV < HT^{ST} \\ \dfrac{HT^{UB} - FV}{HT^{UB} - HT^{ST}}, & \text{if } HT^{ST} \le FV \le HT^{UB} \\ 0, & \text{if } FV > HT^{UB} \end{cases}$$

$$\eta_{SI} = \max\left\{\min\left\{\eta_{L,SI}, \eta_{R,SI}, 1\right\}, 0\right\}$$

FV denotes as a flux of ith reaction or a metabolite flow rate of ith metabolite that is obtained by solving Eq.(S5), and CA^{ST} and HT^{ST} denote as the optimal value of *CA* and *HT* templates obtained from Eq.(S1). The lower bound *LB* and the upper bound *UB* are provided from CA and HT templates as follows: LB = ST/4 and UB = 4ST where *ST* = CA^{ST} (or HT^{ST}), *LB* = CA^{LB} (or HT^{LB}) and *UB* = CA^{UB} (or HT^{UB}).

Figure S4. Two-side membership function to attribute fuzzy dissimilarity and similarity.

The metabolic deviation grade is defined by the mean-min calculation as follows.

$$\eta_{MD} = \left(\left(\eta_{DS} + \eta_{SI}\right)/2 + \min\left\{\eta_{DS}, \eta_{SI}\right\}\right)/2$$

9. The cell mortality, cell viability and metabolic deviation grades for each target are applied to evaluate the decision-making criterion $\eta_D = (\eta_{TR} + \min\{\eta_{TR}, \eta_{CV}, \eta_{MD}\})/2$ in Eq.(S3). The criteria for all targets are then used in the selection and evaluation step of NHDE (Step 5 in Table S2) to reproduce the next better individuals. The

NHDE algorithm carries on iterative crossover, mutation procedures as discussed in the next subsection.

Introduction to Nested Hybrid Differential Evolution (NHDE)

The anticancer target discovery (ACTD) platform can be formulated as a fuzzy multi-objective hierarchical optimization problem. The ACTD platform can be transformed into a maximizing decision-making (MDM) problem by using fuzzy set theory to derive Pareto solutions as shown in Figure S1. The existence and limitation of the transformation have proved in Wang, et al. (2021).

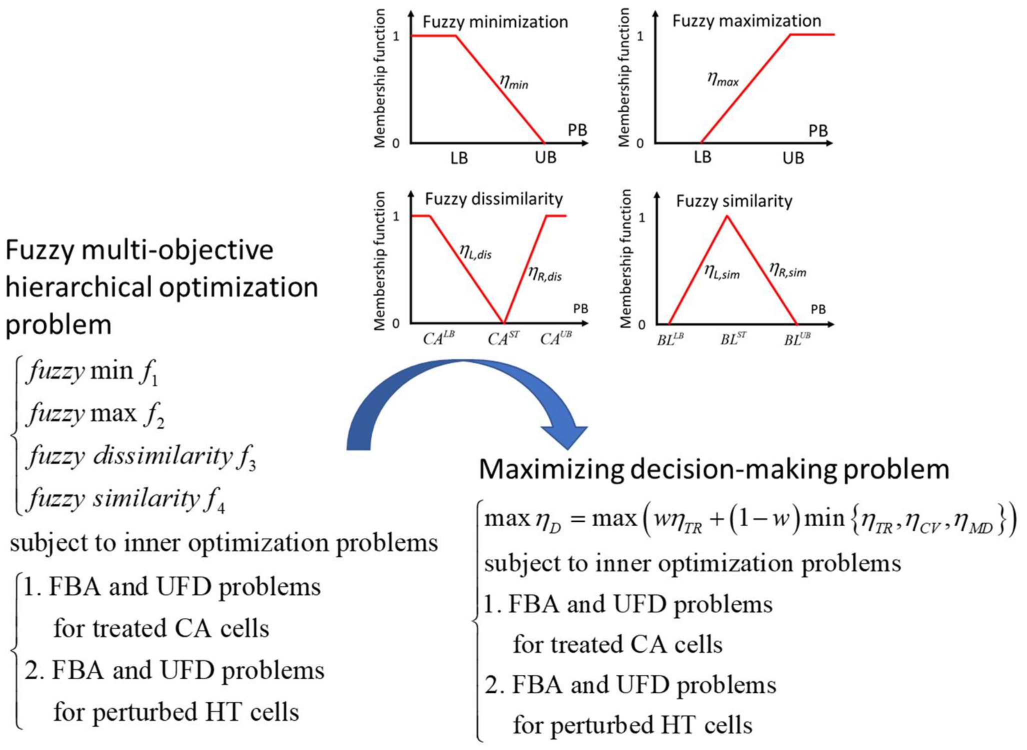

Figure S5. Description of the mathematical strategy for solving a fuzzy multiobjective hierarchical optimization problem.

The MDM problem is rewritten as the following simplified formulation for easily explaining the NHDE algorithm.

$$\begin{cases} \text{Outer optimization problem:} \\ \quad \max f(\mathbf{x},\mathbf{z}) \\ \text{subject to the inner optimization problems:} \\ \begin{cases} \text{Linear programming (LP) problem} \\ \max\limits_{\mathbf{x}} obj = \mathbf{c}^T\mathbf{x} \\ \text{subject to} \\ \quad \mathbf{Ax} = \mathbf{0} \\ \quad \mathbf{x}_{LB} \le \mathbf{x} \le \mathbf{x}_{UB}, \mathbf{z} \notin \Omega^{MU} \\ \quad \mathbf{x}_{LB}^{MU} \le \mathbf{x} \le \mathbf{x}_{UB}^{MU}, \mathbf{z} \in \Omega^{MU} \end{cases} \begin{cases} \text{Quadratic programming (QP) problem} \\ \min\limits_{\mathbf{x}} \mathbf{x}^T\mathbf{x} \\ \text{subject to} \\ \quad \mathbf{Ax} = \mathbf{0} \\ \quad \mathbf{x}_{LB} \le \mathbf{x} \le \mathbf{x}_{UB}, \mathbf{z} \notin \Omega^{MU} \\ \quad \mathbf{x}_{LB}^{MU} \le \mathbf{x} \le \mathbf{x}_{UB}^{MU}, \mathbf{z} \in \Omega^{MU} \\ \quad \mathbf{c}^T\mathbf{x} \ge \mathbf{c}^T\mathbf{x}^* \end{cases} \end{cases}$$

The inner optimization problem consists of LP and QP problems, which is a sequential relationship.

The NHDE algorithm is a stochastic optimization based on hybrid differential evolution (Chiou, 1997), which was extended from the original DE algorithm (Storn and Price, 1996; Storn and Price, 1997). The basic operations of original DE and modified NHDE are shown in Tab. S1. The detailed procedures have discussed by Wang (2017).

Table S1. Basic operations for the original DE and NHDE algorithms

**Original DE**	**Modified NHDE**
1. Representation and initialization	1. Representation and initialization
2. Mutation	2. Mutation with rounding operation
3. Crossover operation	3. Crossover operation
4. Selection and evaluation	4. Restriction operation
5. Repeat steps 2 to 4	5. Selection and evaluation
	6. Solve LP/QP problems for each candidate gene
	7. Compute fitness for each feasible design
	8. Migration operation performed naturally or enforced if necessary
	9. Repeat steps 2 to 6

The computational procedures of NHDE are listed in Tab. S2. NHDE is a parallel direct search algorithm (as shown in Figure S6) that utilizes a population of Np individuals (enzymes) to find an optimal solution. The initialization process randomly generates Np individuals to cover the entire search space uniformly. Each individual in the population consists of a set of enzymes that are selected to be modulated.

The mutation operator of NHDE adopted from DE was an essential component compared with other evolutionary algorithms. Different from conventional evolutionary algorithms, the mutation operation of DE/NHDE uses the difference between two or four randomly chosen individuals as an evolutionary direction. The ith mutant individual $(\mathbf{z}^G)_i$ in generation G is obtained through the difference of two or four random individuals as expressed in the following form:

$$(\mathbf{z}^G)_i = \mathrm{INT}\left\{(\mathbf{z}^G)_p + \rho^G\left[(\mathbf{z}^G)_j - (\mathbf{z}^G)_k + (\mathbf{z}^G)_l - (\mathbf{z}^G)_m\right]\right\}, i = 1,\ldots,N_p$$

where random indices $j,k,l,m \in \{1,\ldots,N_p\}$ are mutually different. The operator INT in the equation is used to rounding the real vector into the integer vector. In DE, the differential mutation factor $\rho^G \in [0, 1.2]$ is fixed and set by the user to obtain faster convergence. This factor is used to control the step length along the searching direction. A random mutation factor was used in NHDE to obtain more diversified individuals. NHDE also includes an additional mutation strategy that applying a linear crossover for the ith individual and the best individual $(\mathbf{z}^G)_b$ to generate the parent individual. The parent individual is therefore expressed as follows:

$$(\mathbf{z}^G)_p = \rho_p^G(\mathbf{z}^G)_b + (1-\rho_p^G)(\mathbf{z}^{G-1})_i$$

where the factor ρ_p^G is a random number between zero and one generated by a uniform distribution generator, and $(\mathbf{z}^{G-1})_i$ indicates the ith mutant individual in the previous generation. The mutation operation may cause the mutant individual escape from the search domain. The mutation operation may cause the mutant individual to escape the search domain (i.e., bounds are violated). If this occurs, it is replaced by a random number within the lower and upper bounds of the particular decision variable, thus restricting to the search domain. The choice of mutation factor for DE/NHDE is heuristic and random. When population diversity is low, candidate individuals rapidly cluster together such that the individuals cannot be further improved, and premature convergence occurs. Similar to conventional evolutionary algorithms, the local population diversity could be increased by using a crossover operation such as a binomial crossover.

NHDE use the difference between two or four mutually independent individuals to determine the direction of search and obtain a mutant individual. This differential mutation converges quickly so that most individuals cluster around the best candidate individual in some generations. Consequently, the population diversity and exploration capability diminish and clustered individuals are unable to reproduce more diversified individuals through the mutation operation because the weighted difference is nearly zero. The recombination of mutant individuals and their clustered parents further prevents the reproduction of a diversified population. Therefore, all individuals quickly cluster together and superior individuals cannot be generated through mutation and crossover operations.

The migration operation of the NHDE algorithm is used to help individuals escape from the local cluster, but this operation is performed only if the population diversity falls below a desired level. The degree of population diversity ζ is introduced to check whether the migration operation should be performed. Each element of the *i*th individual $(\mathbf{z}^G)_i$ in generation G is referred to as a gene of the individual, and the gene diversity index dz_{ji} is given by

$$dz_{ji} = \begin{cases} 0, & \text{if } z_{ji}^G = z_{jb}^G,\ j = 1,\ldots,n; i = 1,\ldots,N_p; i \neq b \\ 1, & \text{otherwise,} \end{cases}$$

where z_{ji}^G and z_{jb}^G are the *j*th gene of the ith and best individual at the *G*th generation, respectively. dz_{ji} is set to zero if the *j*th gene of the *i*th individual is identical to the best gene; otherwise it is set to one (Chiou and Wang, 1999; Liao, et al., 2001). ζ is defined as the ratio of total gene diversities to the total number of genes other than those of the best individual:

$$\zeta = \frac{\sum_{i=1, i\neq b}^{N_p} \sum_{j=1}^{n} dz_{ji}}{n(N_p - 1)}$$

The value of population diversity degree ranges between zero and one. A value of zero implies that all of the genes are clustered around the best individual. On the other hand, a value of one indicates that current candidate individuals are a completely diversified population. The desired tolerance for population diversity is assigned by the user. A tolerance value of zero implies that the migration operation in NHDE is switched off, and one implies that the migration operation is performed at every generation. Consequently, the user can set a tolerance value for population diversity degree, $\varepsilon \in (0, 1)$. If ζ is smaller than ε, then NHDE performs migration operations to regenerate a new population in order to escape from a local point; otherwise, NHDE suspends the migration operation and maintains a constant search direction toward finding a new solution.

Table S2. The NHDE algorithm for iteratively selecting a set of candidate enzymes and to infer optimal oncogenes.

NHDE

---

1. Representation and initialization

$$(\mathbf{z}^0)_i = \text{uniformInt}(\mathbf{z}^{\min}, \mathbf{z}^{\max}),\ i = 1,\ldots, N_p$$

Each individual is generated by an integer random number between $\mathbf{z}_{\min}$ and $\mathbf{z}_{\max}$ with uniform distribution

2. Mutation with rounding operation

$$(\hat{\mathbf{z}}^G)_i = \text{INT}\left\{(\mathbf{z}^G)_p + \rho^G\left[(\mathbf{z}^G)_j - (\mathbf{z}^G)_k + (\mathbf{z}^G)_l - (\mathbf{z}^G)_m\right]\right\}$$

3. Crossover operation

$$z_{ji}^G = \begin{cases} z_{ji}^{G-1}, & \text{if a random number} > C_R \\ \hat{z}_{ji}^G, & \text{otherwise}, j = 1,\ldots,n; i = 1,\ldots,N_p \end{cases}$$

4. Restriction operation

$$z_{ji}^G = \begin{cases} z_{ji}^G, z_{ji}^G \in [z_j^{\min}, z_j^{\max}] \\ \text{uniformInt}(z_j^{\min}, z_j^{\max}), z_{ji}^G \notin [z_j^{\min}, z_j^{\max}] \end{cases}$$

5. Selection and evaluation
   (a) For each enzyme, solve the inner LP/QP problem by LP/QP solver, respectively
   (b) Compute fitness for each feasible solution

   $$fitness = f(\mathbf{x}, \mathbf{z}) + \text{penalty}$$

6. Migration operation performed naturally or enforced if necessary

$$(\mathbf{z}^G)_i = \text{uniformInt}(\mathbf{z}^{\min}, \mathbf{z}^{\max}), \text{if } \zeta \le \varepsilon = [0,1]$$

7. Repeat steps 2 to 6

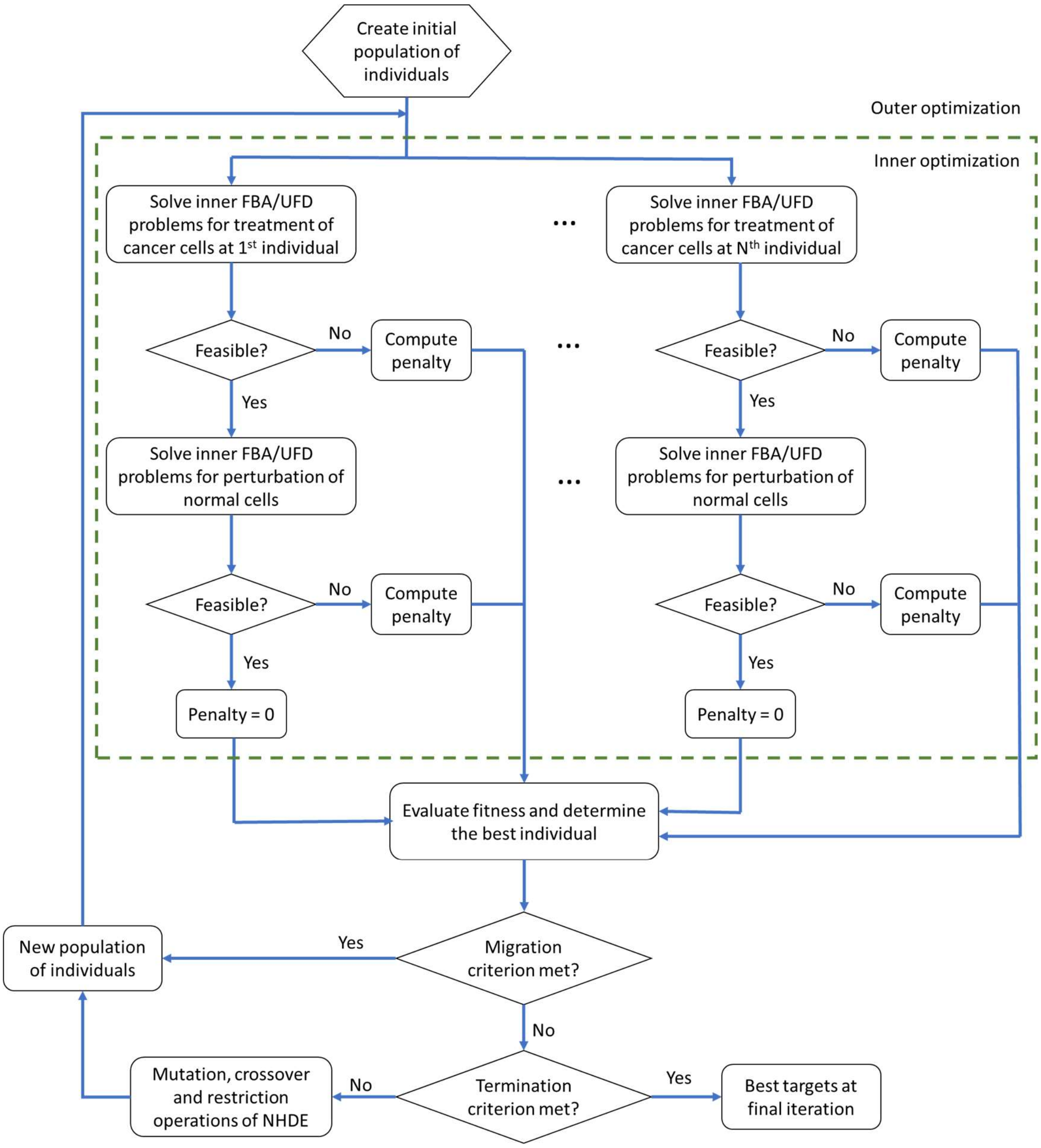

Figure S6. Flowchart of the parallel search algorithm in NHDE

References

Wang, F.S., Wang, T.Y. and Wu, W.H. (2022) Fuzzy multiobjective hierarchical optimization with application to identify antienzymes of colon cancer cells, Journal of the Taiwan Institute of Chemical Engineers, 132, 10412. (doi.org/10.1016/j.jtice.2021.10.021)

Chiou, J.P. and Wang, F.S. (1999) Hybrid method of evolutionary algorithms for static and dynamic optimization problems with application to a fed-batch fermentation process, Computers & Chemical Engineering, 23, 1277-1291. (doi.org/10.1016/S0098-1354(99)00290-2)

Storn, R. and Price, K. (1996) Minimizing the real functions of the ICEC'96 contest by differential evolution. Evolutionary Computation, 1996., Proceedings of IEEE International Conference on. IEEE, Nagoya, pp. 842 - 844. (doi:10.1109/ICEC.1996.542711)

Storn, R. and Price, K. (1997) Differential evolution - A simple and efficient heuristic for global optimization over continuous spaces, Journal of Global Optimization, 11, 341-359. (doi.org/10.1023/A:1008202821328)

Wang, F.S. (2017) Nested differential evolution for mixed-integer bi-level optimization for genome-scale metabolic networks, Ch.12, in Differential evolution in chemical engineering edited by Rangaiah and Sharma, World Scientific. (doi.org/10.1142/10379)
