# Supplementary material for: Identifying essential genes in genome-scale metabolic models of consensus molecular subtypes of colorectal cancer
Source: PLoS One. 2023 May 19;18(5):e0286032. doi: 10.1371/journal.pone.0286032 (PMC10198572; doi:10.1371/journal.pone.0286032)
Supplement: Attachment — Submitted filename: Comments to the Author-v3.docx [file pone.0286032.s008.docx]

**Review Comments to the Author**

Reviewer #1: The authors investigate acquired metabolic sensitivity in different types of colorectal cancer. They use a published definition to assign samples retrieved from the TCGA database into 4 specific and 1 unknown molecular subtype, based on gene expression. They then use this data to reconstruct 6 genome scale metabolic models (5 disease and 1 healthy) and to score their metabolic reactions into 4 confidence levels. They set up a complex hierarchical multi-objective optimization problem to simultaneously estimate the flux in the cells and their acquired sensitivity to knock outs of reactions. This involves solving a flux balance analysis (FBA) problem while minimizing the L2 norm of fluxes weighted by the calculated confidence. The effects of simulated knockouts are graded by their effects on 3 metrics corresponding to 1) the difference in flux between healthy and diseased; 2) the effect on growth and ATP synthesis in the cancer model; 3) the same for healthy model. While the work seems technically sound, it is not clear if its assumptions can be biologically justified and thereby if it has relevance for addressing sensitivity in cancer. Its advantages over current approaches are not quantified.

We thank the reviewer for the accurate summary. The proposed ACTD platform is to mimic a wet-lab process for identifying essential genes. As discussed in the framework of Figure 1, the platform uses flux distribution pattern for cancer (CA) and healthy (HT) cells as the template that refers to as the control in wet-lab processes. A genome-wide flux pattern is not available up-to-date. Therefore, the CA and HT template is obtained through the computation of flux balance analysis and uniform flux distribution problems without consideration of dysregulated restriction. However, the ACTD platform is not only for identifying anticancer targets, but also to evaluate treatment effort.

Major comments
The approach relies on gene expression data to decide on reaction inclusion. While this is commonly done, it should be noted that genes must be translated to proteins for reactions to occur and differences in protein stability and other effects may influence protein levels so that mRNA levels do not accurately reflect them. Furthermore, an expression level below the limit of detection does not necessarily imply that a protein is inactive, since catalytic efficiency varies by orders of magnitude. For tumor samples, genes expression levels may be strongly influenced by the presence of infiltrating immune cells, so observed differences between healthy- and diseased samples may not necessarily reflect changes in the colon cells. It may be appropriate to discuss these limitations in the data used.

The gene expression data of cancer tissue may be infiltrated by immune cells. The expression of genes in immune cells can be different from that in the cancer cells themselves. As a result, it is difficult to identify true changes in gene expression that are specific to the cancer cells, rather than reflecting changes in the immune cells. In this study, we retrieved RNA-seq expression data of colon cancer cells and healthy cells from the TCGA database. The RNA-seq expression data of cancers may be infiltrated by immune cells. However, these data do not explain whether they are infiltration. We did not recognize influent effects of immune cell infiltration, directly used these RNA-seq expressions to reconstruct each corresponding CMS-specific genome-scale metabolic model and the healthy counterpart.

Genome scale metabolic models are underdetermined and there is a large range of flux distributions that may satisfy an objective function. The predicted fluxes are therefore dependent on the modeling choices. Often minimization of the L1 norm (parsimonious FBA) is used to select a unique solution. In this work a weighted L2 norm is used. It is based on the 4 confidence levels derived from gene expression data and they are weighted with factors (0.25, 0.5, 0.75, 1). This weighing is a modeling decision, and it could be of interest to show how the results would be affected by different choices. In particular since the difference between healthy and diseased fluxes, is used in the evaluation and may be affected. One alternative to quantized confidence scores would be a continuous value.

We have discussed the influence effects of the weighting factors assigned and added two tables (S2 Table) in the supplementary in the revised manuscript. The revised part is summarized as follows.

“The computational results may be affected by different weighting factors assigned Eq. (6). In this study, we also considered two additional weighting factors, i.e. the weighting factors based on gene expression levels from zero to one and equal weighting factors, to compute the corresponding flux distributions, and compared them with that using Eq.(7). The ACTD platform used the DMEM medium and different weighting factors to identify a set of single essential genes as shown in S2 Table, respectively. The results reveal that the identified essential genes are irrespective to the assigned weighting factors (S2 Table). However, the cell mortality grades and metabolic deviation grades are different a little. An essential gene is influenced by the topology of a metabolic network, that is a stoichiometric matrix reconstructed from gene expression data. However, weighting factors could make to obtain different flux values, not influence the network structure.”

Table S1. The below table is retrieved from Table 3 in the revised manuscript. The cell mortality grades and metabolic deviation grades for essential genes of each consensus molecular subtype are computed by using DMEM medium and the weighting factors in Eq.(7) of this study.

**Table S2.** The cell mortality grades and metabolic deviation grades for essential genes for each CMS are computed by the weighting factors based on gene expression levels from zero to one, i.e. $c_{k}^{CA/HT}=\left(G_{\max}-G\right)/\left(G_{\max}-G_{\min}\right)$, where G, G_max_ and G_min_ are the level of the regulated gene expression, maximum and minimum gene expression level of the dataset.

**Table S3.** The cell mortality grades and metabolic deviation grades for essential genes for each CMS are computed by the equal weighting factors, i.e. $c_{k}^{CA/HT}=1$.

The objective function for healthy cells is set to maximize ATP synthesis. It is unclear if this is a realistic assumption about what constitutes a healthy state. Furthermore, in absence of limitations on uptake reactions and other constraints, FBA will increase the flux through the objective function until it reaches one of the artificial bounds on internal fluxes (often -1000 or 1000). Is this occurring in these simulations or is this prevented somehow. Are the predicted fluxes reasonable for human cells? Fluxes of around 13.649 mmol/gDW h, are reported, is this level in line with published values for healthy colon cancer cells? It could also be interesting to comment on the difference between culture medium and the in vivo microenvironment, where perhaps metabolites, such as cholesterol, may be present.

The selection of an appropriate objective function is still a challenge in constraint-based metabolic modelling. A common assumption in systems microbiology is that a microorganism tends to optimize its metabolic network in order to maximize the biomass growth rate. It allows for a wide range of predictions consistent with an experimental observation. Moreover, this objective function can be still used to describe cancer cells to divide and proliferate. The objective function used for healthy cells is typically different from that used for cancer cells because the metabolic behavior of healthy cells is distinct from that of cancer cells. ATP is the main source of energy in cells, and maximizing the production of ATP can be used as an objective function to simulate the metabolic behavior of healthy cells. The Recond3D provides the default bounds are not between -1000 and 1000 for all fluxes, but some fluxes between -1 and 1000. In the present study, we also assigned some uptake fluxes in a realizable rang, which are available in the supplementary file at <http://doi.org/10.5281/zenodo.7136561>.

The abstract discusses that gene essentiality prediction is a time-consuming process. However, a brute force method is used in this study to validate the essentiality predictions. It would be useful to quantify the speed up that can be achieved with the proposed approach.

We have discussed the situation in the Result section of the revised manuscript, and summarized as follows.

“With 3.7 GHz processor and 64 GB RAM on an i9 local computer, the enumeration method demanded ~8 CPU hours, while the NHDE algorithm ~6 CPU hours.”

“Conducting an enumerative search for identifying combinations of two target essential genes is time consuming because it requires computations to be performed for more than 4,800,000 combinations for each CMS. We are difficult to use an enumerative search for identifying combinations of two targets. The ACTD platform can be used to reduce the computational burden associated with performing evolutionary procedures.”

In table 4 the cell mortality grade is reported. It appears to be strikingly similar for all genes within each molecular subtype. Is there an explanation to this, e.g. how the objective function is set up. For the metabolic deviation grade metric, the fluxes of the cancer cell model is compared with the healthy flux distribution. Is it always the same flux distribution for the healthy or does it vary between models? It is stated that one objective is to maximize the cell viability of perturbed healthy cells during gene knockout or deletion. Why is it assumed that healthy cells will be resistant to perturbations?

We applied the ACTD platform for identifying essential genes that conduct termination of cell growth when the genes of cancer cells are deleted. Meanwhile, the present study supposes that the essential genes are also inactive on their healthy cells to maintain their cell viability and smaller metabolic alterations. Table 3 of the revised manuscript shows the cell mortality grades of the identified essential genes. The cell mortality grade is computed by the mean-min operation using the defined membership functions of cell growth rate and ATP production rate for treated CA cells.

We have added the 30 tables in the supporting information (see S2 Table) of the revised manuscript to explain the computational results for cell growth rate and ATP production rate for treated CA (TR) cells and perturbed HT (PH) cells. One of 30 tables is shown as below for your evaluation.

CMS1 Model using DMEM medium

The cell mortality grade for each essential gene is computed by using attribution of fuzzy set theory in Eq.(9) of the revised manuscript as follows:

$$\begin{matrix} \eta_{\min}=\left\{\begin{matrix} 1,\text{ if }FV<LB \\ \frac{UB-FV}{UB-LB},\text{ if }LB\leq FV\leq UB \\ 0,\text{ if }FV>UB\text{ } \end{matrix}\right. \\ \eta_{\max}=\left\{\begin{matrix} 0,\text{ if }FV<LB \\ \frac{FV-LB}{UB-LB},\text{ if }LB\leq FV\leq UB \\ 1,\text{ if }FV>UB\text{ } \end{matrix}\right. \end{matrix}$$

We introduce a mean-min operation to compute the cell mortality grade as

$$\eta_{TR}=\left(\left(\eta_{ATP}^{TR}+\eta_{biomass}^{TR}\right)/2+\min\left\{\eta_{ATP}^{TR},\eta_{biomass}^{TR}\right\}\right)/2$$

The biomass growth rate of TR cells is 1.0E-8, and ATP production rates are about 17.2 (see above table) so that the cell mortality grades for all essential genes achieve nearly identical.

The model makes predictions about gene essentiality and the predictions are compared with observations in DepMap. It would be useful to quantify the prediction performance, e.g. in the form of a confusion matrix (with true and false positives) and to apply an appropriate statistical tests. It could also be interesting to speculate on what may be the reason that some predicted essential genes were not essential in DepMap.

The twelfth (N/D) column in Table 3 is experimental data retrieved from DepMap (https://depmap.org/portal/). 54 colon cancer cell lines (D = 54) are used in the experiment test, and to account for the number (N) of the cancer cell lines to be death. In the present study, we do not use the gene expression data for each cancer cell lines to build its corresponding cell-specific genome-scale metabolic model, and then to predict essential genes. Therefore, we are unable to compute four outcomes of a confusion matrix.

What was the rational for perturbing essential genes together with non-essential?

Conducting an enumerative search for identifying combinations of two target essential genes is time consuming because it requires computations to be performed for more than 4,800,000 combinations for each CMS. We are difficult to use an enumerative search for identifying combinations of two targets. The ACTD platform can be used to reduce the computational burden associated with performing evolutionary procedures. We developed the NHDE algorithm with two candidate groups in the ACTD platform for identifying gene combinations. The first candidate group comprised the union set of single essential genes obtained from Table 3, and the second candidate group comprised the other genes from non-essential genes. The computational results (as shown in S4 Table) reveal that optimal combination of two targets can be obtained from the first candidate group, i.e. two single essential genes, and achieved from the first candidate group (a single essential gene) and second candidate group (a non-essential gene). The results revealed that the metabolic deviation grades for most two-target combinations (S4 Table) were superior to those for their corresponding single target essential genes (S3 Table) and that each combination involved at least one essential gene.

Minor comments
It is hard to tell from the methods if any work was done in this study to classify the TCGA samples. If the classification from Guinney 2015 was used without modification, then the corresponding methods section could be simplified.

In the present study, we retrieved the CMS classification data for the CRC samples of TCGA from the literature [Guinney J, Dienstmann R, Wang X, De Reynies A, Schlicker A, et al. The consensus molecular subtypes of colorectal cancer. Nature Medicine. 2015; 21, 1350-1356. doi: 10.1038/nm.3967]. We have omitted the discussion of the classification in the methods in the revised manuscript.

Table 3 does not appear to be showing what was intended, since the first 2 lines for each species show the exact same value, even though there seems to be multiple unique entities for each model.

We have drawn Table 3 to a bar chart as Figure 3 in the revised manuscript to clarify the meaning of the data for the models.

Fig 3. Number of metabolites, reactions, genes, and feasible enzymes for each consensus molecular subtype and its healthy counterpart.

It implied that cells with 0 growth rate are dead, however cells can also enter a state of senescence where they neither grow nor die.

In the present study, the minimum cell growth rate is 1.0E-8 that implies cells is to terminate growth, and may enter maintenance state. The computational result is infeasible if the cell growth rate achieves zero value.

It is mentioned that “inhibition of formate production reduced intracellular cholesterol synthesis and thereby eliminated the growth of cancer cells”. Why is growth eliminated by a mere reduction in biosynthesis rate?

We have revised the sentences to clarify the meaning, and summarized as follows:

“Formate is a crucial molecule in one-carbon metabolism that it serves as an intermediate in is a series of biochemical reactions, including the biosynthesis of cholesterol, nucleotides and amino acids [56]. Specifically, formate is used as a one-carbon donor in the conversion of acetyl-CoA to mevalonate, which is a key intermediate in the cholesterol biosynthetic pathway. In the present study, the inhibition of formate production rate reduced intracellular cholesterol synthesis and thereby eliminated the growth of cancer cells.”

The use of reaction identifiers specific to the recon model, such as ‘R_OMPDC’ may perhaps not be very informative to most readers.

We have added name of each reaction in Table 4 and S6 Table to explain each symbol of reaction.

Perhaps the sentence "Formate is an intermediate metabolite in one-carbon metabolism, and acts as the source and sink in mammalian cell metabolism" may need further clarification about what is considered source and sink.

We have revised the sentences to clarify the meaning, and summarized as follows:

“Formate is a crucial molecule in one-carbon metabolism that it serves as an intermediate in is a series of biochemical reactions, including the biosynthesis of cholesterol, nucleotides and amino acids [56].”
